# Simplification of Carbon Bond Mechanism IV (CBM-IV) under Different Initial Conditions by Using Concentration Sensitivity Analysis

**DOI:** 10.3390/molecules24132463

**Published:** 2019-07-04

**Authors:** Le Cao, Simeng Li, Ziwei Yi, Mengmeng Gao

**Affiliations:** Key Laboratory for Aerosol-Cloud-Precipitation of China Meteorological Administration, Nanjing University of Information Science and Technology, Nanjing 210044, China

**Keywords:** CBM-IV, mechanism simplification, concentration sensitivity analysis

## Abstract

Carbon Bond Mechanism IV (CBM-IV) is a widely used reaction mechanism in which VOCs are grouped according to the molecular structure. In the present study, we applied a sensitivity analysis on the CBM-IV mechanism to clarify the importance of each reaction under two different initial conditions (urban and low-NO_x_ scenarios). The reactions that exert minor influence on the reaction system are then screened out from the mechanism, so that a reduced version of the CBM-IV mechanism under specific initial conditions can be obtained. We found that in a typical urban condition, 11 reactions can be removed from the original CBM-IV mechanism, and the deviation is less than 5% between the results using the original CBM-IV mechanism and the reduced mechanism. Moreover, in a low-NO_x_ initial condition, two more reactions, both of which are nitrogen-associated reactions, can be screened out from the reaction mechanism, while the accuracy of the simulation is still maintained. It is estimated that the reduction of the CBM-IV mechanism can save 11–14% of the computing time in the calculation of the chemistry in a box model simulation.

## 1. Introduction

Air pollution is becoming more severe at many parts of the world, mainly due to increasing human activities such as the burning of fossil fuels [1]. Many types of pollutants such as carbon monoxide (CO) and sulfur dioxide (SO_2_) as well as small particles of soot are formed from the incomplete burning of hydrocarbons in power plants or factories and then released into the ambient air, leading to poor air quality in cities and a damage to the health of the people. For example, it was reported by Chan and Yao [2] that the air quality in mega cities of China exceeds the Chinese Grade-II standard on 10–30% of days based on a few years of data, and the occurrence of ozone pollution events also becomes more frequent due to the increased emission of VOCs. It was also reported by the World Health Organization (WHO) that the annual mean levels of the particulate matter (PM) have increased by more than 8% from 2010 to 2015 based on the data collected in approximately 800 cities around the world [3]. Moreover, it was estimated by WHO [3] that in the year 2012, about 3 million deaths were attributed to air pollution from particulate matter. It is now well known that these pollution events are usually caused by an occurrence of a series of chemical reactions in the atmosphere. For example, the photochemical smog occurred in the troposphere arises from an excessive ozone (O_3_) generated by the oxidation of VOCs in the presence of OH. Thus, in order to understand the principles of the pollution events, formulate control strategies, and solve environmental problems, an accurate description of the complex atmospheric chemical processes is crucial. Atmospheric reaction mechanism, which is an essential part of the air quality model, is a means of representing the atmospheric chemistry quantitatively. By using the atmospheric reaction mechanism, we can not only discover the conversion between the primary pollutants (e.g., VOCs and NO_2_) and the secondary pollutants (e.g., O_3_) [4], but also evaluate the contribution of each reaction to the pollution processes by solving the differential equations based on the chemical reactions included in the mechanism [5]. In recent years, as the knowledge of the atmospheric chemistry goes deeper, a large number of the atmospheric reaction mechanisms such as CBM [6,7,8,9] and SAPRC [10,11,12] were proposed for the investigation of the tropospheric chemical processes.

The Carbon Bond Mechanism (CBM) is a condensed reaction mechanism that is currently used in many photochemical smog or air quality models. In the CBM, VOCs are classified based on the molecular structure. The earliest CBM model, namely CBM-I, contains only four carbon bond types and 32 reactions. However, it can still successfully reproduce the results obtained in smog chamber experiments. After that, based on a large amount of the experimental data, CBM-I was improved extensively, resulting in several versions of CBM [6,7,8,9]. One of these CBM mechanisms, the CBM-IV was proposed by Gery et al. [6], and it contains 33 species and 81 reactions. In CBM-IV, carbon bonds are divided into different functional groups such as paraffin carbon bond (PAR), olefinic carbon bond (OLE) and toluene (TOL). In accompany with the proposal of the CBM-IV mechanism, it was validated by approximately 170 sets of data obtained in UNC and UCR smog chamber experiments [13,14,15,16], and it was found that the simulations with the implementation of the CBM-IV mechanism are in good agreement with the experimental data. Due to the small number of the chemical reactions and the species included in the mechanism, CBM-IV is convenient to be applied in air quality models. Therefore, at present, it is still implemented in many numerical models such as WRF-Chem (Weather Research and Forecasting model coupled to Chemistry) [17] and KPP (Kinetic PreProcessor) [18,19].

Many studies with the implementation of the CBM-IV mechanism were performed. Kang et al. [20] applied CBM-IV into a multi-scale air quality simulation platform, and investigated the association between the O_3_ production and the NO_x_ reduction in airsheds with high $\mathrm{VOCs}/{\mathrm{NO}}_{x}$ ratios. Matthes et al. [21] coupled the CBM-IV mechanism into a circulation model system to study the global impact of road traffic emissions on the tropospheric ozone in the year 1990, and they found that the non-methane hydrocarbon (NMHC) emissions play an important role in changing the global distribution of ozone through the formation and the transport of PAN. Wang et al. [22] investigated the influence of biogenic emissions on the tropospheric ozone in China by using an updated version of CBM-IV in a mesoscale meteorological model (MM5). It was suggested by Wang et al. [22] that the biogenic emissions may have different influence on the change of the tropospherc ozone in different regions, depending on whether the ozone formation in that region is VOC-limited or NO_x_-limited. Oshima et al. [23] developed a box model with the use of CBM-IV to study the extent to which the black carbon particles [24] are coated by other components of aerosols. Observational data such as the size distribution of the black carbon particles obtained from aircraft measurements were also used to evaluate the performance of their model. In recent years, CBM-IV was adopted in simulating the dispersion and photochemical evolution of reactive pollutants in street canyons with the assistance of computational fluid dynamic (CFD) models [25,26,27,28].

Although there exist a large number of numerical studies using the CBM-IV mechanism as mentioned above, the efficiency of the simulation especially the 3-D computations is still strongly limited, mainly due to the difficulties in solving the stiff Jacobi matrix caused by the complex chemical system. It is because that the reaction rates in the atmosphere vary greatly. As a result, the size of the time step used in the estimation of the contribution from chemistry should be much smaller than that used in updating the meteorological field. Therefore, the calculation speed of the whole computing is heavily restricted. It was estimated that more than 95% of the computing time is consumed in the calculation of the chemistry. Moreover, many researchers try to improve the accuracy of the mechanism by adding more reactions, which also adds the complexity of the chemical system and the cost of the computing time. Thus, it is needed to make a simplification of the reaction mechanism under specific initial conditions while the accuracy of the simulations is still retained.

Therefore, in the present study, we applied a concentration sensitivity analysis on the CBM-IV mechanism to identify important reactions in the mechanism under different initial conditions (typical urban and low-NO_x_ scenarios). Then, based on the results of the concentration sensitivity analysis, we removed the reactions that were indicated unimportant for the change of all the chemical species. By doing that, a reduced version of the CBM-IV mechanism can be obtained, which is able to save the computing time under the specific initial conditions. Moreover, the accuracy of the original CBM-IV mechanism can be kept.

The structure of the manuscript is as following. In the next section (Section 2), the method for the simplification of the CBM-IV mechanism and the configurations of the computations are given. In Section 3, most of the computational results are shown, and a comparison between the results of the simulations by using the original CBM-IV mechanism and the reduced one is made. In the last section (Section 4), major conclusions of this paper are drawn, and an extension of the present study is discussed.

## 2. Mathematical Models and Methods

The strategy of the present study is as following. At first, a box model KINAL [29] was used to capture the temporal evolution of the chemical species included in CBM-IV under different initial conditions. The obtained mixing-ratio curves over time were also compared with that obtained by using another open-sourced software KPP [18,19] for the purpose of validation. After verifying the correctness of the KINAL simulations, we continued to perform a concentration sensitivity analysis on the CBM-IV mechanism, followed by a mechanism simplification. By doing that, we were able to obtain a reduced version of the CBM-IV mechanism with less reactions compared to the original one. These procedures were also made by using the box model KINAL. At last, after the simplification of the mechanism, the reduced version of the CBM-IV mechanism was again implemented in KINAL so that a comparison between the results achieved before and after the simplification can be made.

In the following, the details of the model used in the present study as well as the configurations of the computations are given.

### 2.1. Reaction System

In the present study, the change of each chemical species in the reaction system can be described as:

(1) $$\frac{d\vec{c}}{dt}=f\left(\vec{c},\vec{k}\right)+\vec{E}.$$

In Equation (1), $\vec{c}$ denotes a column vector of the species concentrations, and ${c|}_{t=0}=c_{0}$ stands for the initial conditions. The variables, $\vec{k}$, *t* and $\vec{E}$, in Equation (1) represent a vector of reaction rate constants, time, and the local surface emissions, respectively. By solving Equation (1), we were able to capture the change of each species over time. As the reaction rates in the CBM-IV mechanism differ a lot, the Jacobi matrix formed during the process of solving Equation (1) is stiff which adds the difficulty in finding a trade-off between the efficiency and the accuracy. In this study, Equation (1) was solved in a box model KINAL [29]. KINAL is an open-sourced program package written in FORTRAN language, aiming for the simulation of chemical kinetic mechanisms. A subroutine of KINAL, DIFF, can solve stiff equations so that the temporal change of the reaction system can be derived.

After solving Equation (1), we validated the obtained results by using another open-sourced software KPP [18,19]. KPP is a chemical box model for the investigation of dynamic chemical systems, which has been successfully applied in the study of the tropospheric and stratospheric chemistry [18,30,31]. The reason we chose KPP for validation is that many commonly used reaction mechanisms such as CBM-IV and SAPRC-99 [10,11] are originally included in KPP. In our study, we found the deviation between the results of these two different models (KINAL and KPP) with same configurations lower than 1% (see the supplementary material of this manuscript and also the reference [32]), which ensures the correctness of the KINAL computations and enables us for a further concentration sensitivity analysis.

### 2.2. Concentration Sensitivity Analysis

After validating the KINAL results, we continued to conduct a concentration sensitivity analysis on CBM-IV to reduce the size of the mechanism under different initial conditions. In the concentration sensitivity analysis, the importance of the *j*-th reaction for the *i*-th component can be identified by the relative concentration sensitivity coefficient $\tilde{S_{ij}}$. It is expressed as:

(2) $$\tilde{S_{ij}}=\frac{\partial \ln c_{i}}{\partial \ln k_{j}}=\frac{k_{j}}{c_{i}}\frac{\partial c_{i}}{\partial k_{j}}=\frac{k_{j}}{c_{i}}S_{ij}.$$

In Equation (2), $c_{i}$, $k_{j}$ represent the concentration of the *i*-th constituent, and the rate constant of the *j*-th reaction, respectively. $S_{ij}=\partial c_{i}/\partial k_{j}$ denotes the absolute concentration sensitivity, of which the unit depends on the order of the *j*-th reaction. To compare the value of sensitivity coefficients belonging to different reactions, the normalized sensitivity coefficient, $\tilde{S_{ij}}$, is introduced by multiplying $S_{ij}$ with $c_{i}/k_{j}$, so that $\tilde{S_{ij}}$ is a non-dimensional variable. The relative concentration sensitivity $\tilde{S_{ij}}$ can indicate the relative change of the *i*-th species concentration when a small perturbation occurs on the rate constant of the *j*-th reaction.

In this study, $\tilde{S_{ij}}$ can be calculated by taking the partial derivative of the *i*-th constituent in Equation (1) over $k_{j}$. When the local surface emission $\vec{E}$ in Equation (1) is assumed independent on the rate constant, Equation (1) becomes:

(3) $$\frac{d(\partial c_{i}/\partial k_{j})}{dt}=\sum_{l=1}^{n_{s}}\frac{\partial f_{i}}{\partial c_{l}}\frac{\partial c_{l}}{\partial k_{j}}+\frac{\partial f_{i}}{\partial k_{j}},$$

of which the upper limit $n_{s}$ signifies the total number of chemical species included in the mechanism. By replacing $\partial c_{i}/\partial k_{j}$ and $\partial c_{l}/\partial k_{j}$ in Equation (3) with the absolute concentration sensitivity $S_{ij}$ and $S_{lj}$, another form of Equation (3) can be obtained:

(4) $$\frac{dS_{ij}}{dt}=\sum_{l=1}^{n_{s}}\frac{\partial f_{i}}{\partial c_{l}}S_{lj}+\frac{\partial f_{i}}{\partial k_{j}}.$$

The second term on the right-hand side of Equation (4) denotes the direct change of the *i*-th species concentration caused by the change of the *j*-th reaction rate. In contrast to that, the first term on the right-hand side of Equation (4) indicates the indirect effect brought about by the change of the *j*-th reaction rate. After solving Equation (4), the relative sensitivity can be derived by multiplying the absolute concentration sensitivity $S_{ij}$ with $k_{j}/c_{i}$. The subroutine SENS in KINAL can calculate a matrix of the absolute concentration sensitivities of the reaction system, which consequently leads to the estimation of the relative sensitivity.

The concentration sensitivity analysis is effective in clarifying the relative importance of a single reaction for a specific component. Accordingly, removing unimportant reactions from the original mechanism can be made. In this research, if the *j*-th reaction fulfills the criterion shown in Equation (5) according to [33], it is then marked as unimportant and thus can be removed.

(5) $$\max|\tilde{S_{ij}\left(t\right)}|\leq 10\%;i=1,\cdots ,n_{s},t=t\left(1\right),\cdots ,t\left(n_{t}\right).$$

$n_{s}$ in Equation (5) is the total number of the chemical components involved, and $n_{t}$ is the total number of time steps in the process of calculation. By using the criterion shown in Equation (5), if the absolute value of the sensitivity belonging to the *j*-th reaction for all the components is less than 10% at every time point, the *j*-th reaction can be regarded as unimportant and thus can be removed from the original mechanism.

### 2.3. Configurations of the Model

In this study, we investigated the simplification of the CBM-IV mechanism under two different initial conditions (urban and low-NO_x_ scenarios), and the initial conditions of the model are shown in Table 1. In the simulation under a typical urban condition, the initial mixing ratio of NO_x_ (NO+NO_2_) is in the order of tens of ppb (ppb = parts per billion). In contrast to that, in the low-NO_x_ case, we reduced the initial NO_x_ amount to 100 ppt (ppt = parts per trillion). Besides, we increased the initial value of HONO from 1 ppb to 30 ppb according to [34]. In the present study, we computed the temporal change of the reaction system within 5 days, and the start time of the simulation is set to 12:00 of the first day.

The reaction mechanism used in the present study is the original version of CBM-IV, which was proposed by Gery et al. [6]. It consists of 33 chemical species and 81 reactions, which are listed in the Appendix A of this manuscript. Generally, four different types of species are included in the CBM-IV mechanism: (1) Inorganic species; (2) Organic species that are explicitly represented due to their unique chemical natures in the environment, such as the formaldehyde (FORM); (3) Organic species denoted by carbon bonds, such as olefins (OLE); (4) Organic species represented by the molecular structure, such as aromatic hydrocarbons (e.g., TOL). The values of the rate constant *k* in the CBM-IV mechanism used in the present simulation are adopted from Gery et al. [6], and can be found in the Appendix A of this manuscript. They are calculated by using the Arrhenius formula [35]:

(6) $$k=Ae^{\frac{-E_{a}}{RT}}.$$

In Equation (6), *R* (unit: $J\;K^{-1}\;{\mathrm{mol}}^{-1}$) is the universal gas constant, and $E_{a}$ (unit: $J\;{\mathrm{mol}}^{-1})$ is the activation energy. *T* (unit: K) is the temperature, and *A* represents the pre-exponential factor that has a same unit as *k*, depending on the order of the reaction. In this study a constant temperature T=288.15 K is assumed, which is the same as the settings in the KPP examples [34].

With respect to the photolytic reactions in the mechanism, according to KPP [18,19], a radian-like parameter $T_{\mathrm{tmp}}$ is introduced to consider the influence on the photolysis rates exerted by the change of the solar zenith angle,

(7) $$T_{\mathrm{tmp}}=\frac{2T_{\mathrm{local}}-T_{\mathrm{sunset}}-T_{\mathrm{sunrise}}}{T_{\mathrm{sunset}}-T_{\mathrm{sunrise}}}.$$

In Equation (7), the sunrise time, $T_{\mathrm{sunrise}}$, is set to 4:30 a.m. in our simulations, and the sunset time $T_{\mathrm{sunset}}$ is defined as 7:30 p.m. $T_{\mathrm{local}}$ in Equation (7) represents the local hours of the day. When $T_{\mathrm{local}}$ resides between 4.5 (the sunrise time) and 19.5 (the sunset time), the photolytic reactions are switched on in the model. Otherwise, they are switched off.

## 3. Results and Discussion

To investigate the simplification of the CBM-IV mechanism under different initial conditions, two simulation scenarios were set up, urban and low-NO_x_ cases. The initial condition of the urban scenario was adopted from the benchmark example [34] originally included in KPP (see Table 1). In contrast to that, in the low-NO_x_ scenario, the initial mixing ratios of NO and NO_2_ were reduced to 100 ppt, much lower than the typical value range (i.e., tens of ppb) used in the urban simulation.

The simulation results under these two scenarios are presented separately below.

### 3.1. Urban Scenario

**Figure 1:**
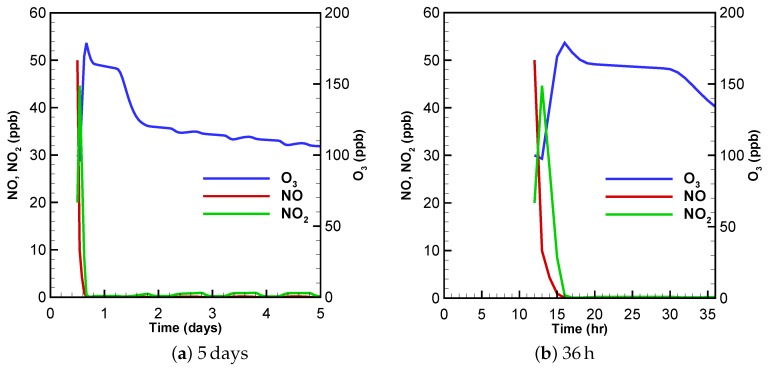
Mixing-ratio profiles as a function of time for O_3_, NO and NO_2_ within (**a**) 5 days and (**b**) 36 h under the typical urban initial condition [32].

In this simulation, the initial air composition contains high levels of pollutants, such as NO_x_, PAN and O_3_ (see Table 1). Figure 1 shows the time behavior of major components, O_3_, NO and NO_2_, under this typical urban condition. These mixing-ratio results over time have been discussed in detail in [32]. Thus, in this paper we only describe them briefly. It is seen in Figure 1a that during the simulated 5 days, the mixing ratio of ozone increases rapidly within the first few hours, from its initial value 100 ppb to a peak value of approximate 180 ppb. After reaching the maximum, the O_3_ level starts to decline due to the photolytic decomposition. When the nighttime comes at 7:30 p.m. of the first day (see Figure 1a), the decrease rate of the O_3_ mixing ratio becomes lower. However, when the sun rises at 4:30 a.m. of the next day, the amount of ozone continues to drop, until the ozone value reaches a relatively stable level when the end of the simulation is approached.

With respect to the temporal evolution of the nitrogen oxides (NO_x_ = NO + NO_2_), it is seen in Figure 1a that the variation of NO and NO_2_ occurs mostly within the first few hours. Thus, we show the change of the NO_x_ mixing ratios during the first 36 h in Figure 1b. It is seen that the mixing ratio of NO decreases monotonously from its initial value to a near-zero value within 4 h (from 12:00 to 16:00 on the first day). In contrast to the NO profile, the mixing ratio of NO_2_ increases at the beginning of the simulation, reaching a peak value of approximately 45 ppb. Then it decreases to less than 1 ppb before hour 16. It should be noted that during the increase of NO_2_, a negative correlation between the mixing ratios of O_3_ and NO_2_ is found. It is because that at the early stage of the simulation, the amount of NO is abundant. The chemical system composed of ozone and nitrogen oxides is thus mainly controlled by the titration reaction:

(R1) $$\mathrm{NO}+O_{3}\to {\mathrm{NO}}_{2}+O_{2},$$

leading to an enhancement of NO_2_ and a decrease of NO and O_3_. However, because the O_3_ depleted in Reaction (R1) can be compensated by that formed in the OH radical chain reaction of VOCs:

(R2) $$\begin{array}{cc} OH+RH+O_{2}\to & \;RO_{2}+H_{2}O\\ RO_{2}+NO\to & \;RO+NO_{2}\\ NO_{2}+h\nu \to & \;NO+O{(}^{3}P) \end{array}\;\frac{O{(}^{3}P)+O_{2}\overset{M}{\to }\;O_{3}}{\mathrm{Net}:OH+RH+2\;O_{2}\overset{M}{\to }\;RO+O_{3}+H_{2}O,}$$

the loss of O_3_ is thus found much less than the increase of NO_2_ (see Figure 1b). Please note that O(^3^P) in Reaction (R2) denotes the oxygen atom in the ground state. When the reaction proceeds, after hour 16, the amount of NO_x_ given in the initial condition is almost completely consumed due to the conversion of NO_2_ to HNO_3_ and PAN:

(R3) $$NO_{2}+OH\overset{M}{\to }HNO_{3},$$

(R4) $$NO_{2}+C_{2}O_{3}\to PAN.$$

As a result, in this time period, the ozone loss and formation is strongly influenced by the photolytic reaction

(R5) $$O_{3}+h\nu \to O{(}^{1}D)+O_{2},$$

(R6) $$O{(}^{1}D)+H_{2}O\to 2OH,$$

in which O(^1^D) denotes electronically excited state oxygen atom, and the NO_x_ regenerated by the reaction of OH radical with HNO_3_ during the daytime:

(R7) $$OH+HNO_{3}\overset{M}{\to }NO_{3}+H_{2}O,$$

(R8) $$NO_{3}+h\nu \to 0.89NO_{2}+0.89O{(}^{3}P)+0.11NO.$$

**Figure 2:**
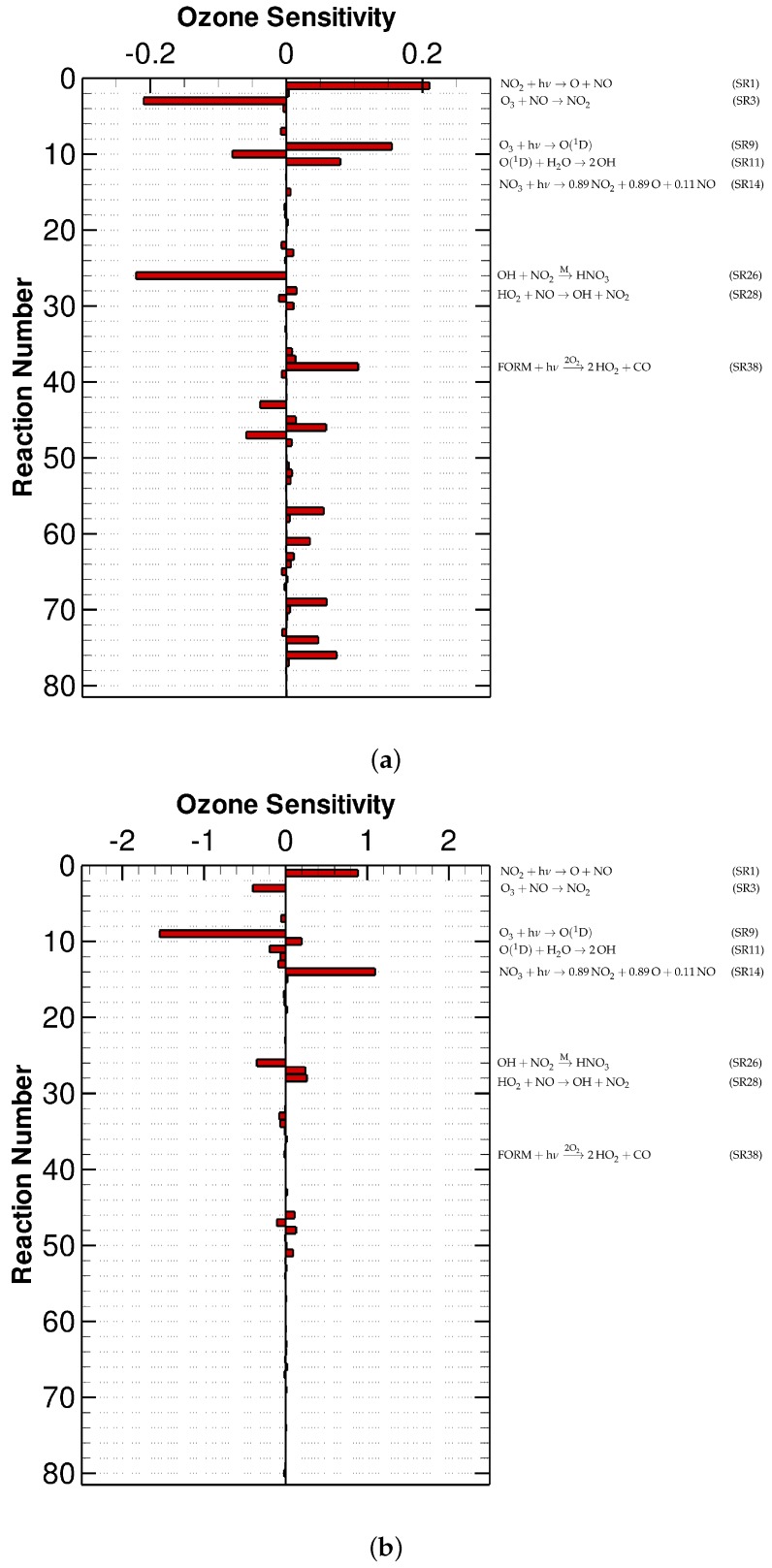
The relative concentration sensitivity of O_3_ in the urban scenario at (**a**) the first hour and (**b**) the 96th hour of the simulation.

Later, we plotted the values of the ozone sensitivity coefficient corresponding to each reaction in the CBM-IV mechanism, at the time points representing the beginning (the 1st hour) and the end of the simulation (the 96th hour, i.e., 12:00 of the 5th day) (see Figure 2). It is seen in Figure 2a that at the beginning of the simulation, the values of the ozone sensitivity for all the reactions in the mechanism are mostly less than 0.2. Among these reactions, the most dominant reactions at this time are Reactions (SR1) $NO_{2}+h\nu \to O{(}^{3}P)+NO$, (SR3) $O_{3}+NO\to NO_{2}$ and (SR26) $OH+NO_{2}\overset{M}{\to }HNO_{3}$ (see the Appendix A for the index of the reactions). Among these three reactions, it is not surprising that Reactions (SR1) and (SR3) have large influence on the change of the ozone concentration, as these two reactions are parts of the reaction cycle between the nitrogen oxides and ozone. Aside from this, the ozone mixing ratio at this time has the strongest negative dependence on Reaction (SR26), as this reaction is able to convert OH and NO_2_ to HNO_3_, leading to a major depletion of ozone during this time period as mentioned above. These findings again suggest that at this time stage, the ozone mixing ratio is mostly determined by the titration reaction and the high initial values of the nitrogen oxides.

When the end of the simulation is approached, the ozone sensitivity to each reaction in the mechanism changes (see Figure 2b). In general, most of the O_3_ sensitivities increase as the simulation proceeds, denoting an enhanced dependence of the ozone mixing ratio on the local chemistry. It should be noted that Reactions (SR9) and (SR11)

(SR9) $$O_{3}+h\nu \to O{(}^{1}D),$$

(SR11) $$O{(}^{1}D)+H_{2}O\to 2OH,$$

have a negative influence on the production of O_3_ at the 96th hour (see Figure 2b), while at the first hour these sensitivity coefficients are positive. It is because that at the beginning of the simulation when the amount of NO_x_ is abundant, the photolysis of ozone leads to a formation of OH and a following OH radical chain reaction of VOCs as mentioned above. As a result, the photolytic decomposition of ozone promotes the formation of ozone. In contrast to that, when the end of the 5-day simulation comes, as the nitrogen oxides are almost completely consumed, the amount of ozone formed from the OH radical chain reaction is less than the loss of ozone caused by the photolysis of ozone, leading to a change in the sign of the sensitivities corresponding to these two reactions. Moreover, because the initial nitrogen oxides are mostly converted to HNO_3_ and PAN at this end time, ozone is mainly consumed by its photolytic reaction, i.e., Reaction (SR9), instead of the HNO_3_ formation reaction (SR26). Thus, the dominance of Reaction (SR9) is highlighted during the end of the simulation (see Figure 2b). In addition, we also found the sensitivity value of Reaction (SR38)

(SR38) $$HCHO+h\nu \overset{2O_{2}}{\to }2HO_{2}+CO$$

declines remarkably over time. This is because that the most deterministic factor for the ozone change at this time is the availability of NO_x_ rather than HO_2_.

We then applied the selection criterion described in Equation (5) on the obtained relative concentration sensitivities. Eleven reactions, (SR5), (SR6), (SR20), (SR21), (SR25), (SR40), (SR42), (SR55), (SR56), (SR60) and (SR75) were identified as unimportant so that they can be eliminated from the original reaction mechanism under this urban condition. As a result, a simplified version of the CBM-IV mechanism, consisting of 33 species and 70 reactions, is obtained. We found the change of all the constituents in the simulation using the simplified mechanism is almost identical to that using the original CBM-IV mechanism (not shown here). By comparing the obtained values (see Table 2), we found the maximum deviation between these two results smaller than 5%, which proves the correctness of our mechanism simplification under this urban initial condition. We then estimated the computing time saved by applying the simplified mechanism in KINAL, and it was found that approximately 11% of the computing time can be saved, which enables a faster calculation of the chemistry in applications. However, it should be noted that the exact computing time that can be saved depends on the situation. In our box model computation, the time saved by the mechanism simplification is mainly in the order of minutes to hours, while in 3-D simulations it might be in the order of days or weeks, depending on the mesh resolution and the length of the time step.

### 3.2. Low-NO_x_ Scenario

**Figure 3:**
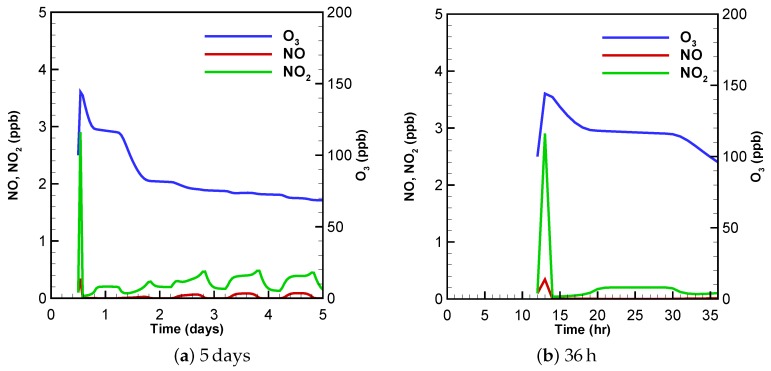
Mixing-ratio profiles as a function of time for O_3_, NO and NO_2_ within (**a**) 5 days and (**b**) 36 h under the low-NO_x_ condition.

In this case, we reduced the initial mixing ratio of the nitrogen oxides (NO from 50 to 0.1 ppb. NO_2_ from 20 to 0.1 ppb), and increased the initial value of HONO to 30 ppb according to [34]. The temporal evolution of ozone, NO and NO_2_ under this low-NO_x_ condition is shown in Figure 3. As seen in Figure 3, the profile change of ozone under these conditions is similar to that in the urban scenario shown in Figure 1. The ozone mixing ratio increases to a peak value within a few hours (see Figure 3a), and then drops to a relatively stable level at the end of the computation. However, the maximum value (∼140 ppb) and the value at the end of the simulation (∼70 ppb) are much lower than those in the urban scenario simulation, 180 ppb and 105 ppb, respectively. These lower ozone values are expected as the formation of ozone is inhibited due to the lack of nitrogen oxides in this low-NO_x_ condition. Moreover, it may also be observed in Figure 3b that under these experimental conditions, the mixing ratio of ozone increases directly from its original value to the peak value, while in the urban scenario the ozone mixing ratio decreases at the beginning (see Figure 1b). The reason for the observed difference may also be attributed to the lower amount of NO and NO_2_ under given experimental conditions so that the formation of O_3_ by the oxidation of VOCs exceeds the loss of O_3_ caused by the titration reaction at the start of the simulation.

Regarding to the nitrogen oxides, it was found that the trends of the NO and NO_2_ change are similar in this low-NO_x_ scenario. Both of these two species mixing ratios increase from the start of the computation, reaching a peak value (2.9 ppb for NO_2_ and 0.3 ppb for NO) at the first hour (see Figure 3b), and then decline to an amount less than 0.1 ppb at about 2 h after the start of the simulation. The maximum value of the NO_2_ mixing ratio in this condition is found remarkably lower than that in the urban scenario, due to the smaller initial value of the nitrogen oxides. Furthermore, different from the urban simulation in which the NO mixing ratio decreases monotonously, in this low-NO_x_ scenario, the value of NO increases at the beginning, due to the photolytic decomposition of the initial HONO at this moment.

**Figure 4:**
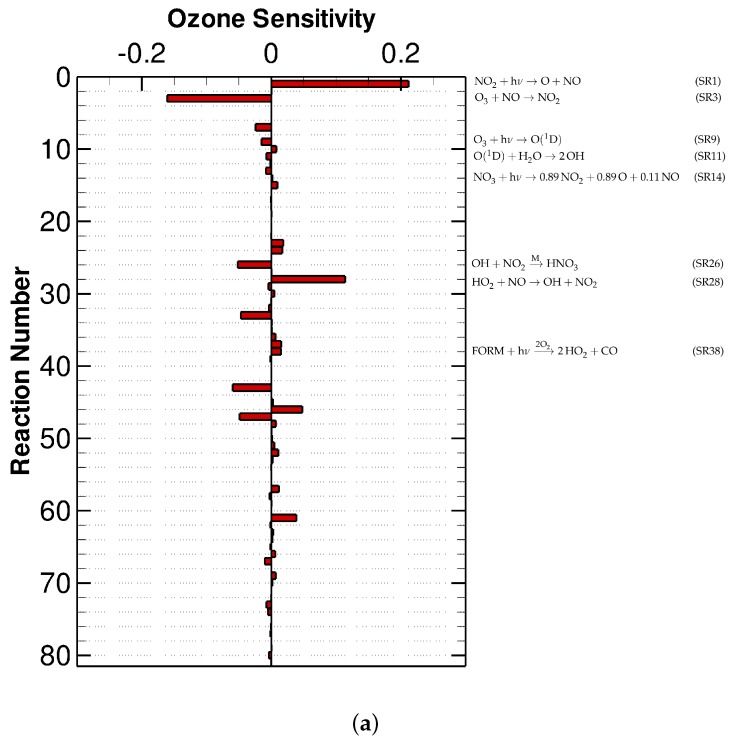

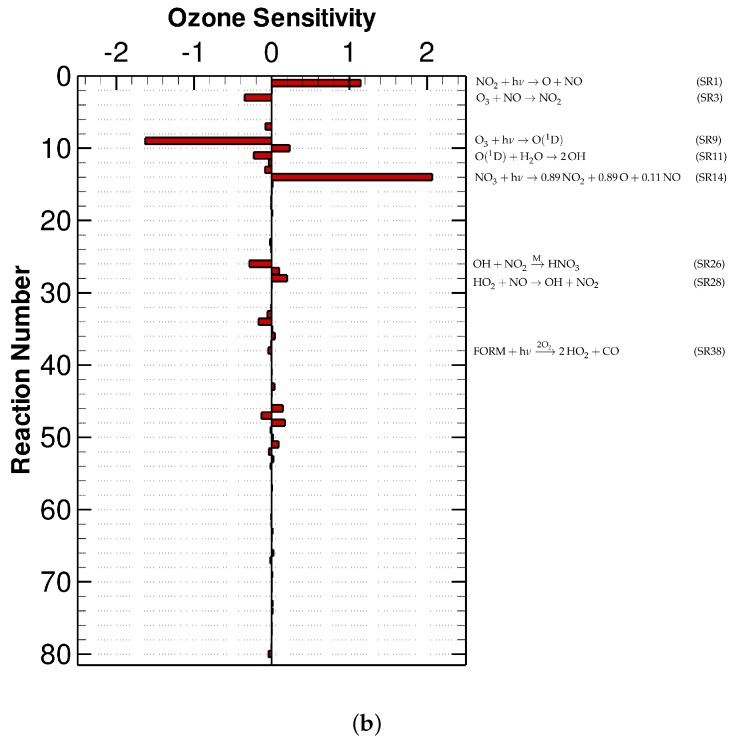
The relative sensitivity of O_3_ in the low-NO_x_ scenario at (**a**) the first hour and (**b**) the 96th hour.

The O_3_ sensitivity values at the first hour and the 96th hour under low-NO_x_ experimental conditions are shown in Figure 4. Similar to the results of the urban case scenario, at the beginning of the simulation, the O_3_ sensitivity is generally lower than 0.2 (see Figure 4a). By comparing Figure 4a with Figure 2a, an enhanced importance of Reaction (SR28) $HO_{2}+NO\to OH+NO_{2}$ is indicated in the low-NO_x_ computation. It is because that when a low value is initially given to the nitrogen oxides, the availability of NO_2_ turns out to be the rate-determining factor for the ozone formation. As a result, the ozone becomes very sensitive to the rate change of Reaction (SR28) that converts NO to NO_2_. In contrast to that, at a later period of this simulation (see Figure 4b), the most important reaction for the formation of O_3_ is Reaction (SR14) $NO_{3}+h\nu \to 0.89NO_{2}+0.89O{(}^{3}P)+0.11NO$. It is because that at this time, the NO_3_ regenerated from Reaction (SR27) $OH+HNO_{3}\overset{M}{\to }NO_{3}$ during the daytime acts as the major source of the nitrogen oxides as discussed above. In comparison with that, the loss of ozone at this time is strongly influenced by Reactions (SR9), (SR11) and (SR26) (see Figure 4b), which is similar to the situation in the urban simulation shown in Figure 2b.

In contrast to the urban scenario, in this low-NO_x_ simulation, apart from the 11 reactions identified as negligible in the urban scenario, two more reactions in the mechanism are indicated as unimportant:

(SR4) $$O{(}^{3}P)+NO_{2}\to NO+O_{2},$$

(SR44) $$ALD_{2}+NO_{3}\overset{O_{2}}{\to }C_{2}O_{3}+HNO_{3}.$$

It was found that during the computation process, the influence of these two reactions on the change of all the constituents in the mechanism is minor, so that they can be eliminated from the mechanism. It was also noticed that both reactions are nitrogen-associated reactions, reflecting a lower importance of the nitrogen related species under this low-NO_x_ initial condition. After removing these 13 reactions indicated in the sensitivity analysis, a reduced reaction mechanism that is composed of 33 species and 68 reactions is obtained under the low-NO_x_ condition. It was observed that the change of all the air components over time by using different reaction mechanisms is similar, denoting a minor difference between the results before and after the simplification procedure. In consistency with the results shown in the previous section, we also see in Table 3 that the deviation of each species mixing ratio is small, with a maximum lower than 3%, which also suggests a successful mechanism reduction. Besides, due to the simplification, it was estimated that 14% of the computing time can be saved in the calculation of the chemistry in this condition.

## 4. Conclusions and Future Work

In this study, we used a concentration sensitivity analysis to study the relative importance of individual reaction in the CBM-IV mechanism under different initial conditions. Reactions that exert negligible impact on the reaction system were eliminated from the mechanism so that a mechanism reduction under specific initial conditions can be made. In the present study, we found that when a typical urban condition is initially given, at the beginning of the simulation, the reaction system especially the ozone mixing ratio is deeply influenced by the titration reaction and the high initial values of nitrogen oxides. However, when the end of the 5-day computation is approached, because the nitrogen oxides are mostly converted to HNO_3_ and PAN, the ozone value in the CBM-IV mechanism depends heavily on its photolytic reaction and the amount of NO_x_ regenerated by the reaction between HNO_3_ and OH. In this condition, we found that 11 reactions are indicated as unimportant and thus can be removed from the original CBM-IV mechanism. The maximum deviation of all the simulated species concentration between the results before and after the simplification was found less than 5%. It was also estimated that the reduction of the mechanism enables a 11% saving of the computing time.

On the contrary, when the initial value of the nitrogen oxides is reduced to 0.1 ppb, the ozone change at an early period of the simulation was found mostly determined by the availability of NO_2_ as well as the reaction converting NO to NO_2_. However, when the end of the low-NO_x_ simulation comes, the production of O_3_ depends mostly on the photolysis of NO_3_, which is similar to the situation in the urban case. It was also found that 13 reactions including two more nitrogen-associated reactions were identified as negligible in this low-NO_x_ scenario compared to the urban scenario, and can be screened out from the original CBM-IV mechanism. By implementing the mechanism after the simplification into the box model KINAL, we were able to save 14% of the computing time, while the deviation between the results before and after the mechanism reduction is lower than 3%.

In the future, we plan to continue our research by investigating more advanced reaction mechanisms such as CB05 [8], CB6 [9] and SAPRC-07 [12], which are used more frequently at present. The full plan is that we first investigate the internal properties of these reaction mechanisms by using the concentration sensitivity analysis. After that, we will make a simplification of these mechanisms based on the results of the sensitivity analysis and analyze the differences between these mechanisms. Observational data obtained from field campaigns or environmental monitoring stations are also needed to evaluate the mechanisms and confirm the findings. Aside from this, it might also be interesting to figure out the common features of the removed reactions under the given initial conditions. Moreover, the impact brought about by the inclusion of the surface emission on the simplification of the mechanism should be studied. It is also useful to extend the method presented in this manuscript to 3-D simulations. For this purpose, we need to compare the timescale of the atmospheric chemistry with that of air transport such as the horizontal advection and the vertical turbulent mixing. At present, the authors are developing a mechanism simplification method in which the influence of air diffusion can be considered.

## Figures and Tables

**Table 1:** Initial conditions used in the simulations of the urban scenario [34] and the low-NO_x_ scenario (unit: ppb, ppb = parts per billion). The species not listed in this table are assumed to have a near-zero initial value ($10^{-5}$ ppt).

Species	Urban	Low-NO_x_
NO	50	0.1
${\mathrm{NO}}_{2}$	20	0.1
HONO	1	30
$O_{3}$	100	100
CO	300	300
HCHO	10	10
ALD2	10	10
PAN	1	1
ETH	10	10
TOL	10	10
XYL	10	10
ISO	10	10
Relative humidity		30%
Temperature		288.15 K
Altitude		0 km
Pressure		1013.25 hPa
Air density		$2.55\times 10^{19}$ molec. cm${}^{-3}$

**Table 2:** The maximum deviation of each species mixing ratio between the results using the original CBM-IV mechanism and the reduced one during the whole urban scenario computation.

Species	Maximum Deviation (%)	Species	Maximum Deviation (%)	Species	Maximum Deviation (%)
NO	3.4	TOL	0.5	PNA	3.1
NO_2_	3.2	XYL	0.8	$C_{2}O_{3}$	2.9
HONO	1.9	ISOP	4.1	MGLY	0.5
O_3_	0.1	H_2_O	0.0	O(^1^D)	0.1
CO	0.0	HO_2_	0.4	O(^3^P)	0.4
FORM	0.2	H_2_O_2_	1.2	CRES	1.0
${\mathrm{ALD}}_{2}$	0.3	OH	0.4	CRO	2.3
PAN	0.4	XO_2_	1.2	NO_3_	1.7
PAR	0.2	ROR	0.2	${\mathrm{TO}}_{2}$	0.4
OLE	0.6	XO_2_N	1.5	OPEN	0.5
ETH	0.4	HNO_3_	0.5	N_2_O_5_	4.6

**Table 3:** The maximum deviation of each species mixing ratio between the results using the original CBM-IV mechanism and the reduced one during the whole low-NO_x_ scenario computation.

Species	Maximum Deviation (%)	Species	Maximum Deviation (%)	Species	Maximum Deviation (%)
NO	1.8	TOL	0.2	PNA	0.4
NO_2_	0.4	XYL	0.3	$C_{2}O_{3}$	0.4
HONO	0.5	ISOP	0.6	MGLY	0.2
O_3_	0.0	H_2_O	0.0	O(^1^D)	0.0
CO	0.0	HO_2_	0.1	O(^3^P)	0.0
FORM	0.0	H_2_O_2_	0.0	CRES	2.2
${\mathrm{ALD}}_{2}$	1.2	OH	0.1	CRO	0.3
PAN	0.0	XO_2_	0.2	NO_3_	2.1
PAR	0.0	ROR	0.1	${\mathrm{TO}}_{2}$	0.2
OLE	0.3	XO_2_N	0.4	OPEN	0.2
ETH	0.5	HNO_3_	0.1	N_2_O_5_	2.1

## Supplementary Materials

The following are available online at https://www.mdpi.com/1420-3049/24/13/2463/s1, Table S1: Peak values of major components (NO_2_, H_2_O_2_, O_3_, CO, NO and PAN) obtained in KPP and KINAL, and the deviation of the peak values between these two models. Figure S1: Temporal change of (a) NO_2_, H_2_O_2_, (b) O_3_, CO, (c) NO and PAN obtained in KPP and KINAL.

## Appendix A

Table A1 gives a detailed listing of the reactions and the rate constants included in the CBM-IV mechanism.

**Table A1:** Carbon Bond Mechanism IV (CBM-IV) listing.

Reaction Number	Reaction	Rate Constant (*k*) (${\mathrm{cm}}^{3}\;\mathrm{molec}.^{-1}\;s^{-1}$)
(SR1)	${\mathrm{NO}}_{2}+h\nu \to O{(}^{3}P)+\mathrm{NO}$	radiation dependent
(SR2)	$O{(}^{3}P)\overset{O_{2},\;M}{\to }O_{3}$	$1.4\times 10^{3}e^{\frac{1175}{T}}$
(SR3)	$O_{3}+\mathrm{NO}\to {\mathrm{NO}}_{2}$	$1.8\times 10^{-12}e^{\frac{1370}{T}}$
(SR4)	$O{(}^{3}P)+{\mathrm{NO}}_{2}\to \mathrm{NO}$	$9.3\times 10^{-12}$
(SR5)	$O{(}^{3}P)+{\mathrm{NO}}_{2}\overset{M}{\to }{\mathrm{NO}}_{3}$	$1.6\times 10^{-13}e^{\frac{687}{T}}$
(SR6)	$O{(}^{3}P)+\mathrm{NO}\overset{M}{\to }{\mathrm{NO}}_{2}$	$2.2\times 10^{-13}e^{\frac{602}{T}}$
(SR7)	$O_{3}+{\mathrm{NO}}_{2}\to {\mathrm{NO}}_{3}$	$1.2\times 10^{-13}e^{\frac{-2450}{T}}$
(SR8)	$O_{3}+h\nu \to O{(}^{3}P)$	radiation dependent
(SR9)	$O_{3}+h\nu \to O{(}^{1}D)$	radiation dependent
(SR10)	$O{(}^{1}D)\overset{M}{\to }O{(}^{3}P)$	$1.9\times 10^{8}e^{\frac{390}{T}}$
(SR11)	$O{(}^{1}D)+H_{2}O\to 2\;\mathrm{OH}$	$2.2\times 10^{-10}$
(SR12)	$O_{3}+\mathrm{OH}\to {\mathrm{HO}}_{2}$	$1.6\times 10^{-12}e^{\frac{-940}{T}}$
(SR13)	$O_{3}+{\mathrm{HO}}_{2}\to \mathrm{OH}$	$1.4\times 10^{-14}e^{\frac{-580}{T}}$
(SR14)	${\mathrm{NO}}_{3}+h\nu \to 0.89\;{\mathrm{NO}}_{2}+0.89\;O{(}^{3}P)+0.11\;\mathrm{NO}$	radiation dependent
(SR15)	${\mathrm{NO}}_{3}+\mathrm{NO}\to 2\;{\mathrm{NO}}_{2}$	$1.3\times 10^{-11}e^{\frac{250}{T}}$
(SR16)	${\mathrm{NO}}_{3}+{\mathrm{NO}}_{2}\to \mathrm{NO}+{\mathrm{NO}}_{2}$	$2.5\times 10^{-14}e^{\frac{-1230}{T}}$
(SR17)	${\mathrm{NO}}_{3}+{\mathrm{NO}}_{2}\overset{M}{\to }N_{2}O_{5}$	$5.3\times 10^{-13}e^{\frac{256}{T}}$
(SR18)	$N_{2}O_{5}+H_{2}O\to 2\;{\mathrm{HNO}}_{3}$	$1.3\times 10^{-21}$
(SR19)	$N_{2}O_{5}\overset{M}{\to }{\mathrm{NO}}_{3}+{\mathrm{NO}}_{2}$	$3.5\times 10^{14}e^{\frac{-10897}{T}}$
(SR20)	$\mathrm{NO}+\mathrm{NO}\overset{O_{2}}{\to }2\;{\mathrm{NO}}_{2}$	$1.8\times 10^{-20}e^{\frac{530}{T}}$
(SR21)	$\mathrm{NO}+{\mathrm{NO}}_{2}+H_{2}O\to 2\;\mathrm{HONO}$	$4.4\times 10^{-40}$
(SR22)	$\mathrm{OH}+\mathrm{NO}\overset{M}{\to }\mathrm{HONO}$	$4.5\times 10^{-13}e^{\frac{806}{T}}$
(SR23)	HONO+hν→OH+NO	radiation dependent
(SR24)	$\mathrm{OH}+\mathrm{HONO}\to {\mathrm{NO}}_{2}$	$6.6\times 10^{-12}$
(SR25)	$\mathrm{HONO}+\mathrm{HONO}\to \mathrm{NO}+{\mathrm{NO}}_{2}$	$1.6\times 10^{-20}$
(SR26)	$\mathrm{OH}+{\mathrm{NO}}_{2}\overset{M}{\to }{\mathrm{HNO}}_{3}$	$1.0\times 10^{-12}e^{\frac{713}{T}}$
(SR27)	$\mathrm{OH}+{\mathrm{HNO}}_{3}\overset{M}{\to }{\mathrm{NO}}_{3}$	$5.1\times 10^{-15}e^{\frac{1000}{T}}$
(SR28)	${\mathrm{HO}}_{2}+\mathrm{NO}\to \mathrm{OH}+{\mathrm{NO}}_{2}$	$3.7\times 10^{-12}e^{\frac{240}{T}}$
(SR29)	${\mathrm{HO}}_{2}+{\mathrm{NO}}_{2}\overset{M}{\to }\mathrm{PNA}$	$1.2\times 10^{-13}e^{\frac{749}{T}}$
(SR30)	$\mathrm{PNA}\overset{M}{\to }{\mathrm{HO}}_{2}+{\mathrm{NO}}_{2}$	$4.8\times 10^{13}e^{\frac{-10121}{T}}$
(SR31)	$\mathrm{OH}+\mathrm{PNA}\to {\mathrm{NO}}_{2}$	$1.3\times 10^{-12}e^{\frac{380}{T}}$
(SR32)	${\mathrm{HO}}_{2}+{\mathrm{HO}}_{2}\to H_{2}O_{2}$	$5.9\times 10^{-14}e^{\frac{1150}{T}}$
(SR33)	${\mathrm{HO}}_{2}+{\mathrm{HO}}_{2}+H_{2}O\to H_{2}O_{2}$	$2.2\times 10^{-38}e^{\frac{5800}{T}}$
(SR34)	$H_{2}O_{2}+h\nu \to 2\;\mathrm{OH}$	radiation dependent
(SR35)	$\mathrm{OH}+H_{2}O_{2}\to {\mathrm{HO}}_{2}$	$3.1\times 10^{-12}e^{\frac{-187}{T}}$
(SR36)	$\mathrm{OH}+\mathrm{CO}\overset{O_{2}}{\to }{\mathrm{HO}}_{2}$	$2.2\times 10^{-13}$
(SR37)	$\mathrm{FORM}+\mathrm{OH}\overset{O_{2}}{\to }{\mathrm{HO}}_{2}+\mathrm{CO}$	$1.0\times 10^{-10}$
(SR38)	$\mathrm{FORM}+h\nu \overset{2O_{2}}{\to }2\;{\mathrm{HO}}_{2}+\mathrm{CO}$	radiation dependent
(SR39)	FORM+hν→CO	radiation dependent
(SR40)	$\mathrm{FORM}+O{(}^{3}P)\to \mathrm{OH}+{\mathrm{HO}}_{2}+\mathrm{CO}$	$3.0\times 10^{-11}e^{\frac{-1550}{T}}$
(SR41)	$\mathrm{FORM}+{\mathrm{NO}}_{3}\overset{O_{2}}{\to }{\mathrm{HNO}}_{3}+{\mathrm{HO}}_{2}+\mathrm{CO}$	$6.3\times 10^{-16}$
(SR42)	${\mathrm{ALD}}_{2}+O{(}^{3}P)\overset{O_{2}}{\to }C_{2}O_{3}+\mathrm{OH}$	$1.2\times 10^{-11}e^{\frac{-986}{T}}$
(SR43)	${\mathrm{ALD}}_{2}+\mathrm{OH}\to C_{2}O_{3}$	$7.0\times 10^{-12}e^{\frac{250}{T}}$
(SR44)	${\mathrm{ALD}}_{2}+{\mathrm{NO}}_{3}\overset{O_{2}}{\to }C_{2}O_{3}+{\mathrm{HNO}}_{3}$	$2.5\times 10^{-15}$
(SR45)	${\mathrm{ALD}}_{2}+h\nu \overset{2O_{2}}{\to }\mathrm{FORM}+{\mathrm{XO}}_{2}+\mathrm{CO}+2\;{\mathrm{HO}}_{2}$	radiation dependent
(SR46)	$C_{2}O_{3}+\mathrm{NO}\overset{O_{2}}{\to }\mathrm{FORM}+{\mathrm{XO}}_{2}+{\mathrm{HO}}_{2}+{\mathrm{NO}}_{2}$	$5.4\times 10^{-12}e^{\frac{250}{T}}$
(SR47)	$C_{2}O_{3}+{\mathrm{NO}}_{2}\to \mathrm{PAN}$	$8.0\times 10^{-20}e^{\frac{5500}{T}}$
(SR48)	$\mathrm{PAN}\to C_{2}O_{3}+{\mathrm{NO}}_{2}$	$9.4\times 10^{16}e^{\frac{-14000}{T}}$
(SR49)	$C_{2}O_{3}+C_{2}O_{3}\to 2\;\mathrm{FORM}+2\;{\mathrm{XO}}_{2}+2\;{\mathrm{HO}}_{2}$	$2.0\times 10^{-12}$
(SR50)	$C_{2}O_{3}+{\mathrm{HO}}_{2}\to 0.79\;\mathrm{FORM}+0.79\;{\mathrm{XO}}_{2}+0.79\;{\mathrm{HO}}_{2}+0.79\;\mathrm{OH}$	$6.5\times 10^{-12}$
(SR51)	$\mathrm{OH}\to \mathrm{FORM}+{\mathrm{XO}}_{2}+{\mathrm{HO}}_{2}$	$1.1\times 10^{2}e^{\frac{-1710}{T}}$
(SR52)	$\mathrm{PAR}+\mathrm{OH}\to 0.87\;{\mathrm{XO}}_{2}+0.13\;{\mathrm{XO}}_{2}N+0.11\;{\mathrm{HO}}_{2}$	$8.1\times 10^{-13}$
	$+0.11\;{\mathrm{ALD}}_{2}+0.76\;\mathrm{ROR}-0.11\;\mathrm{PAR}$	
(SR53)	$\mathrm{ROR}\to 1.10\;{\mathrm{ALD}}_{2}+0.96\;{\mathrm{XO}}_{2}N+0.94\;{\mathrm{HO}}_{2}$	$1.0\times 10^{15}e^{\frac{-8000}{T}}$
	$+0.04\;{\mathrm{XO}}_{2}N+0.02\;\mathrm{ROR}-2.10\;\mathrm{PAR}$	
(SR54)	$\mathrm{ROR}\to {\mathrm{HO}}_{2}$	$1.6\times 10^{3}$
(SR55)	$\mathrm{ROR}+{\mathrm{NO}}_{2}\to$	$1.5\times 10^{-11}$
(SR56)	$O{(}^{3}P)+\mathrm{OLE}\to 0.63\;{\mathrm{ALD}}_{2}+0.38\;{\mathrm{HO}}_{2}+0.28\;{\mathrm{XO}}_{2}$	$1.2\times 10^{-11}e^{\frac{-324}{T}}$
	$+0.30\;\mathrm{CO}+0.20\;\mathrm{FORM}+0.02\;{\mathrm{XO}}_{2}N+0.22\;\mathrm{PAR}$	
	+0.20OH	
(SR57)	$\mathrm{OH}+\mathrm{OLE}\to \mathrm{FORM}+{\mathrm{ALD}}_{2}+{\mathrm{XO}}_{2}+{\mathrm{HO}}_{2}$	$5.2\times 10^{-12}e^{\frac{504}{T}}$
	−PAR	
(SR58)	$O_{3}+\mathrm{OLE}\to 0.50\;{\mathrm{ALD}}_{2}+0.74\;\mathrm{FORM}+0.33\;\mathrm{CO}$	$1.4\times 10^{-14}e^{\frac{-2105}{T}}$
	$0.44\;{\mathrm{HO}}_{2}+0.22\;{\mathrm{XO}}_{2}+0.10\;\mathrm{OH}-\mathrm{PAR}$	
(SR59)	${\mathrm{NO}}_{3}+\mathrm{OLE}\to 0.91\;{\mathrm{XO}}_{2}+\mathrm{FORM}+{\mathrm{ALD}}_{2}$	$7.7\times 10^{-15}$
	$+0.09\;{\mathrm{XO}}_{2}N+{\mathrm{NO}}_{2}-\mathrm{PAR}$	
(SR60)	$O{(}^{3}P)+\mathrm{ETH}\to \mathrm{FORM}+0.70\;{\mathrm{XO}}_{2}+\mathrm{CO}$	$1.0\times 10^{-11}e^{\frac{-792}{T}}$
	$+1.70\;{\mathrm{HO}}_{2}+0.30\;\mathrm{OH}$	
(SR61)	$\mathrm{OH}+\mathrm{ETH}\to {\mathrm{XO}}_{2}+1.56\;\mathrm{FORM}+{\mathrm{HO}}_{2}+0.22\;{\mathrm{ALD}}_{2}$	$2.0\times 10^{-12}e^{\frac{411}{T}}$
(SR62)	$O_{3}+\mathrm{ETH}\to \mathrm{FORM}+0.42\;\mathrm{CO}+0.12\;{\mathrm{HO}}_{2}$	$1.3\times 10^{-14}e^{\frac{-2633}{T}}$
(SR63)	$\mathrm{OH}+\mathrm{TOL}\to 0.08\;{\mathrm{XO}}_{2}+0.36\;\mathrm{CRES}+0.44\;{\mathrm{HO}}_{2}$	$2.1\times 10^{-}1^{2}e^{\frac{322}{T}}$
	$+0.56\;{\mathrm{TO}}_{2}$	
(SR64)	${\mathrm{TO}}_{2}+\mathrm{NO}\to 0.90\;{\mathrm{NO}}_{2}+0.90\;\mathrm{OPEN}+0.90\;{\mathrm{HO}}_{2}$	$8.1\times 10^{-12}$
(SR65)	${\mathrm{TO}}_{2}\to {\mathrm{HO}}_{2}+\mathrm{CRES}$	4.2
(SR66)	$\mathrm{OH}+\mathrm{CRES}\to 0.40\;\mathrm{CRO}+0.60\;{\mathrm{XO}}_{2}+0.60\;{\mathrm{HO}}_{2}$	$4.1\times 10^{-11}$
	+0.30OPEN	
(SR67)	${\mathrm{NO}}_{3}+\mathrm{CRES}\to \mathrm{CRO}+{\mathrm{HNO}}_{3}$	$2.2\times 10^{-11}$
(SR68)	$\mathrm{CRO}+{\mathrm{NO}}_{2}\to$	$1.4\times 10^{-11}$
(SR69)	$\mathrm{OH}+\mathrm{XYL}\to 0.70\;{\mathrm{HO}}_{2}+0.50\;{\mathrm{XO}}_{2}+0.20\;\mathrm{CRES}$	$1.7\times 10^{-11}e^{\frac{116}{T}}$
	$+0.80\;\mathrm{MGLY}+1.10\;\mathrm{PAR}+0.30\;{\mathrm{TO}}_{2}$	
(SR70)	$\mathrm{OH}+\mathrm{OPEN}\to {\mathrm{XO}}_{2}+C_{2}O_{3}+2\;{\mathrm{HO}}_{2}+2\;\mathrm{CO}$	$3.0\times 10^{-11}$
	+FORM	
(SR71)	$\mathrm{OPEN}+h\nu \to C_{2}O_{3}+\mathrm{CO}+{\mathrm{HO}}_{2}$	radiation dependent
(SR72)	$O_{3}+\mathrm{OPEN}\to 0.03\;{\mathrm{ALD}}_{2}+0.62\;C_{2}O_{3}+0.70\;\mathrm{FORM}$	$5.4\times 10^{-17}e^{\frac{-500}{T}}$
	$+0.03\;{\mathrm{XO}}_{2}+0.69\;\mathrm{CO}+0.08\;\mathrm{OH}+0.76\;{\mathrm{HO}}_{2}$	
	+0.20MGLY	
(SR73)	$\mathrm{OH}+\mathrm{MGLY}\to {\mathrm{XO}}_{2}+C_{2}O_{3}$	$1.7\times 10^{-11}$
(SR74)	$\mathrm{MGLY}+h\nu \to C_{2}O_{3}+\mathrm{CO}+{\mathrm{HO}}_{2}$	radiation dependent
(SR75)	$O{(}^{3}P)+\mathrm{ISOP}\to 0.60\;{\mathrm{HO}}_{2}+0.80\;{\mathrm{ALD}}_{2}+0.55\;\mathrm{OLE}$	$1.8\times 10^{-11}$
	$+0.50\;{\mathrm{XO}}_{2}+0.50\;\mathrm{CO}+0.45\;\mathrm{ETH}+0.90\;\mathrm{PAR}$	
(SR76)	$\mathrm{OH}+\mathrm{ISOP}\to \mathrm{FORM}+{\mathrm{XO}}_{2}+0.67\;{\mathrm{HO}}_{2}$	$9.6\times 10^{-11}$
	$+0.40\;\mathrm{MGLY}+0.20\;C_{2}O_{3}+\mathrm{ETH}+0.20\;{\mathrm{ALD}}_{2}$	
	$+0.13\;{\mathrm{XO}}_{2}N$	
(SR77)	$O_{3}+\mathrm{ISOP}\to \mathrm{FORM}+0.40\;{\mathrm{ALD}}_{2}+0.55\;\mathrm{ETH}$	$1.2\times 10^{-17}$
	$+0.20\;\mathrm{MGLY}+0.06\;\mathrm{CO}+0.10\;\mathrm{PAR}+0.44\;{\mathrm{HO}}_{2}$	
	+0.10OH	
(SR78)	${\mathrm{NO}}_{3}+\mathrm{ISOP}\to {\mathrm{XO}}_{2}N$	$3.2\times 10^{-13}$
(SR79)	${\mathrm{XO}}_{2}+\mathrm{NO}\to {\mathrm{NO}}_{2}$	$8.1\times 10^{-12}$
(SR80)	${\mathrm{XO}}_{2}+{\mathrm{XO}}_{2}\to$	$1.7\times 10^{-14}e^{\frac{1300}{T}}$
(SR81)	${\mathrm{XO}}_{2}N+\mathrm{NO}\to$	$6.8\times 10^{-13}$

## References

[1] Akimoto H. (2003). Global air quality and pollution. Science.

[2] Chan C.K., Yao X. (2008). Air pollution in mega cities in China. Atmos. Environ..

[3] WHO (2016). Ambient Air Pollution: A Global Assessment of Exposure and Burden of Disease.

[4] Dodge M.C. (2000). Chemical oxidant mechanisms for air quality modeling: Critical review. Atmos. Environ..

[5] Jimenez P., Baldasano J.M., Dabdub D. (2003). Comparison of photochemical mechanisms for air quality modeling. Atmos. Environ..

[6] Gery M.W., Whitten G., Killus J.P., Dodge M.C. (1989). A photochemical kinetics mechanism for urban and regional scale computer modeling. J. Geophys. Res..

[7] Zaveri R.A., Peters L.K. (1999). A new lumped structure photochemical mechanism for large-scale applications. J. Geophys. Res. Atmos..

[8] Yarwood G., Rao S., Yocke M., Whitten G. (2005). Updates to the Carbon Bond Chemical Mechanism: CB05.

[9] Yarwood G., Jung J., Whitten G., Heo G., J M., M E. Updates to the Carbon Bond Mechanism for Version 6 (CB6). Proceedings of the 9th Annual CMAS Conference.

[10] Carter W.P. (2000). Documentation of the SAPRC-99 Chemical Mechanism for VOC Reactivity Assessment.

[11] Carter W.P. (2000). Implementation of the SAPRC-99 Chemical Mechanism into the Models-3 Framework.

[12] Carter W.P. (2010). Development of the SAPRC-07 chemical mechanism. Atmos. Environ..

[13] Pitts J.N., Darnall K., Carter W., Winer A., Atkinson R. (1977). Mechanisms of Photochemical Reactions in Urban Air.

[14] Jeffries H.E., Kamens R.M., Sexton K.G., Gerhardt A.A. (1982). Outdoor Smog Chamber Experiments to Test Photochemical Models.

[15] Jeffries H.E., Sexton K.G., Kamens R.M., Holleman M.S. (1985). Outdoor Smog Chamber Experiments to Test Photochemical Models: Phase II.

[16] Carter W.P.L., Dodd M.C., Long W.D., Atkinson R., . Dodge M.C. (1985). Outdoor Chamber Study to Test Multi-Day Effects.

[17] Grell G.A., Peckham S.E., Schmitz R., McKeen S.A., Frost G., Skamarock W.C., Eder B. (2005). Fully coupled “online” chemistry within the WRF model. Atmos. Environ..

[18] Damian V., Sandu A., Damian M., Potra F.A., Carmichael G.R. (2002). The kinetic preprocessor KPP—A software environment for solving chemical kinetics. Comput. Chem. Eng..

[19] Sandu A., Sander R. (2006). Technical note: Simulating chemical systems in Fortran90 and Matlab with the Kinetic PreProcessor KPP-2.1. Atmos. Chem. Phys..

[20] Kang D., Aneja V.P., Mathur R., Ray J.D. (2004). Observed and modeled VOC chemistry under high VOC/NO conditions in the Southeast United States national parks. Atmos. Environ..

[21] Matthes S., Grewe V., Sausen R., Roelofs G.J. (2007). Global impact of road traffic emissions on tropospheric ozone. Atmos. Chem. Phys..

[22] Wang Q., Han Z., Wang T., Zhang R. (2008). Impacts of biogenic emissions of VOC and NOx on tropospheric ozone during summertime in eastern China. Sci. Total Environ..

[23] Oshima N., Koike M., Zhang Y., Kondo Y., Moteki N., Takegawa N., Miyazaki Y. (2009). Aging of black carbon in outflow from anthropogenic sources using a mixing state resolved model: Model development and evaluation. J. Geophys. Res. Atmos..

[24] Liu C., Chung C.E., Yin Y., Schnaiter M. (2018). The absorption Ångström exponent of black carbon: from numerical aspects. Atmos. Chem. Phys..

[25] Garmory A., Kim I., Britter R., Mastorakos E. (2009). Simulations of the dispersion of reactive pollutants in a street canyon, considering different chemical mechanisms and micromixing. Atmos. Environ..

[26] Kwak K.H., Baik J.J. (2012). A CFD modeling study of the impacts of NOx and VOC emissions on reactive pollutant dispersion in and above a street canyon. Atmos. Environ..

[27] Kwak K.H., Baik J.J., Lee K.Y. (2013). Dispersion and photochemical evolution of reactive pollutants in street canyons. Atmos. Environ..

[28] Kwak K.H., Baik J.J. (2014). Diurnal variation of NOx and ozone exchange between a street canyon and the overlying air. Atmos. Environ..

[29] Turányi T. (1990). KINAL—A program package for kinetic analysis of reaction mechanisms. Comput. Chem..

[30] Sandu A., Daescu D.N., Carmichael G.R. (2003). Direct and adjoint sensitivity analysis of chemical kinetic systems with KPP: Part I—Theory and software tools. Atmos. Environ..

[31] Daescu D.N., Sandu A., Carmichael G.R. (2003). Direct and adjoint sensitivity analysis of chemical kinetic systems with KPP: II—Numerical validation and applications. Atmos. Environ..

[32] Cao L., Gao M., Li S., Yi Z., Meng X. (2019). Sensitivity analysis of the dependence of the carbon bond mechanism IV (CBM-IV) on the initial air composition under an urban condition. Atmos. Environ..

[33] Cao L., Wang C., Mao M., Grosshans H., Cao N. (2016). Derivation of the reduced reaction mechanisms of ozone depletion events in the Arctic spring by using concentration sensitivity analysis and principal component analysis. Atmos. Chem. Phys..

[34] Sandu A., Verwer J., Loon M.V., Carmichael G., Potra F., Dabdub D., Seinfeld J. (1997). Benchmarking stiff ode solvers for atmospheric chemistry problems-I. implicit vs explicit. Atmos. Environ..

[35] Atkins P., de Paula J. (2010). Atkins’ Physical Chemistry.

