# Supplementary material for: Simplification of Carbon Bond Mechanism IV (CBM-IV) under Different Initial Conditions by Using Concentration Sensitivity Analysis
Source: Molecules. 2019 Jul 4;24(13):2463. doi: 10.3390/molecules24132463 (PMC6651397; doi:10.3390/molecules24132463)
Supplement: Supplementary file 1 [file molecules-24-02463-s001.pdf]

Supplementary Materials: Simplification of Carbon Bond Mechanism IV (CBM-IV) under Different Initial Conditions by Using Concentration Sensitivity Analysis

Le Cao *, Simeng Li, Ziwei Yi and Mengmeng Gao

Comparison of KINAL and KPP Simulations

Figure A1 shows the temporal changes of six components (NO, NO_2, H_2O_2, O_3, CO and PAN) calculated by KPP and KINAL, and Tab. A1 lists the maximum mixing-ratio of these components and the deviation of these peak values between KPP and KINAL. It can be seen that the mixing-ratio profiles obtained in these two different models are nearly identical, and the maximum deviation of these peak values is less than 1%. Thus, the change of the chemical species in these two models is consistent, which validates the correctness of the KINAL computations applying CBM-IV mechanism. Therefore, we can use KINAL further to investigate the internal properties of the CBM-IV mechanism.

Table A1. Peak values of major components (NO_2, H_2O_2, O_3, CO, NO and PAN) obtained in KPP and KINAL, and the deviation of the peak values between these two models.

	Peak Value in KINAL (unit: ppb)	Peak Value in KPP (unit: ppb)	Deviation
NO_2	44.63	44.62	0.02%
H_2O_2	44.58	44.57	0.02%
O_3	178.80	178.29	0.29%
CO	366.20	366.07	0.04%
NO	50	50	0%
PAN	29.74	29.75	0.03%

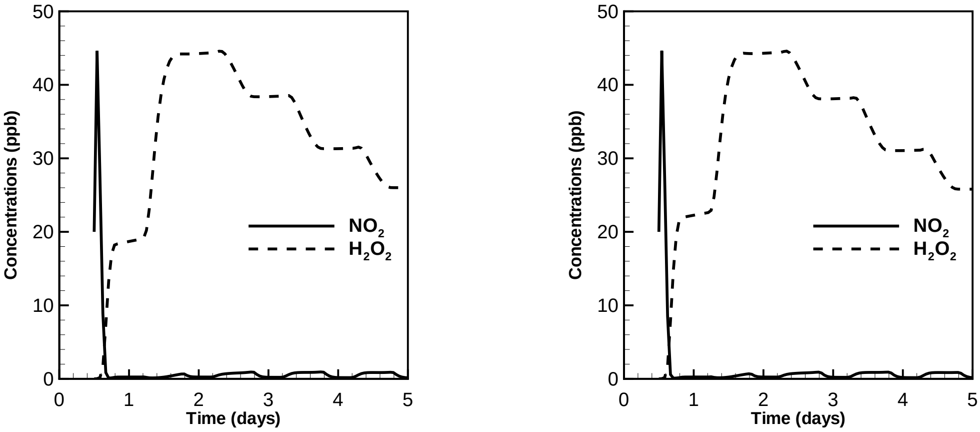

(a)

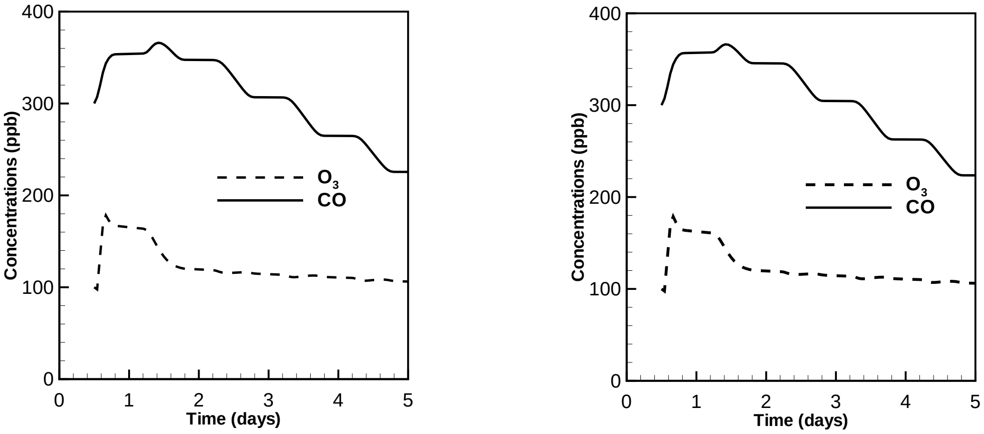

(b)

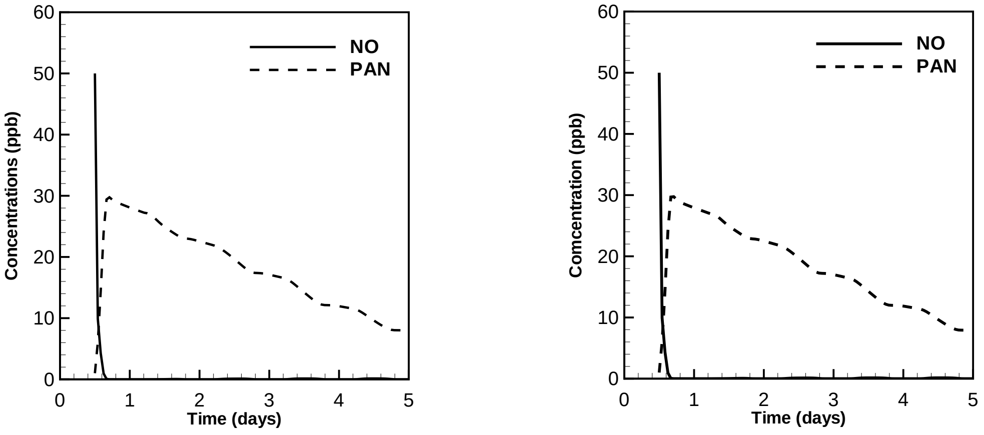

(c)

Figure A1. Temporal change of (a) NO_2, H_2O_2, (b) O_3, CO, (c) NO and PAN obtained in KPP and KINAL. The figures on the left column show the results of KPP, and the right column denotes the results of KINAL.
